# Predicting economic activity using atmospheric nitrogen dioxide (NO2) satellite data: Evidence from local economic indicators in Japan

**DOI:** 10.1371/journal.pone.0337901

**Published:** 2025-12-04

**Authors:** Stefaniia Parubets, Hisahiro Naito

**Affiliations:** Graduate School of Humanities and Social Sciences, University of Tsukuba, Tsukuba, Japan; National Autonomous University of Mexico Institute of Geophysics: Universidad Nacional Autonoma de Mexico Instituto de Geofisica, MEXICO

## Abstract

Accurate and timely measurement of subnational economic activity is crucial for policymakers during economic crises, natural disasters and pandemics such as COVID-19. The availability of such measurement enables policymakers to identify affected regions quickly, allocate emergency resources efficiently, and target fiscal interventions. Satellite-based indicators such as nighttime lights data can be used for such purposes. Nighttime lights data are now widely used to measure economic activity, yet recent studies have highlighted several limitations, including saturation in densely populated areas, omission of daytime activity, inconsistencies among satellite sensors, and measurement errors in regions without electrification. To address these issues, this study evaluates nitrogen dioxide (NO₂) as an alternative satellite-based indicator of regional economic activity in Japan. NO₂, primarily emitted from combustion processes in transportation and industry, provides a direct measure of economic production that complements nighttime lights data. Using two-way fixed-effects panel regressions, we examine the relationship between NO₂ concentrations and prefectural gross domestic product across multiple sectors. At a spatial resolution of 0.25 degrees (0.25°), NO₂ concentrations exhibit statistically and economically significant associations with gross domestic product across most sectors, with particularly strong relationships in energy-intensive industries. However, when higher-resolution data (0.1 degrees (0.1°)) are used, most coefficients lose statistical significance, and some reverse sign in ways that contradict theoretical expectations. These results highlight both the advantages of using NO₂ over nighttime lights data for measuring subnational economic activity and the importance of appropriate spatial scale. Our findings suggest that moderate-resolution satellite data may more accurately capture regional economic patterns than finer-resolution alternatives, provided the data are properly calibrated.

## Introduction

Timely and geographically precise measurement of economic activity is essential for accurate economic analysis and effective policymaking. However, traditional indicators such as gross domestic product (GDP) have significant limitations for area-specific or near real-time analysis, as they typically provide only national-level data at low temporal frequency.

To address these limitations, researchers have increasingly used satellite-derived nighttime lights data, particularly in regions where conventional data are limited or official statistics are unreliable. For example, a widely cited study by Henderson et al. (2012) links real GDP growth to changes in satellite-based nighttime lights intensity. An analysis of panel data from 188 countries between 1992 and 2008 finds that a 1% increase in nighttime lights intensity corresponds, on average, to a 0.3% increase in measured GDP [1].

Nonetheless, researchers have identified several drawbacks in using nighttime lights data to measure economic activity. In countries with well-developed statistical systems, nighttime luminosity tends to be less informative because official economic data are already accurate and comprehensive [2]. Calibration inconsistencies across satellite sensors can introduce measurement errors, particularly when combining data from different sources or time periods [3]. In densely populated urban areas, sensor saturation prevents the detection of brightness variation, limiting the ability of nighttime lights data to capture differences in economic development [4]. Conversely, in rural regions with low population density, nighttime lights data often fail to reflect the full extent of economic activity, potentially leading to inaccurate assessments [5].

One might argue that carbon dioxide, which is released during fossil fuel combustion, could serve as an alternative indicator to nighttime lights for monitoring economic activity. However, using carbon dioxide for this purpose presents substantial challenges for several reasons. First, carbon dioxide remains in the atmosphere for hundreds to even hundreds of thousands of years [6]. Consequently, its concentration reflects the cumulative buildup of emissions over many decades rather than the emissions produced within a single year. Second, due to the diffuse and persistent nature of carbon dioxide, its measurement is subject to considerable uncertainty. Third, because of its long atmospheric lifetime, it is difficult to relate local carbon dioxide concentrations directly to contemporaneous or regional economic activity.

A more suitable alternative for measuring economic activity is to use NO₂, which is primarily produced by fossil fuel combustion in sectors such as manufacturing, transportation, and energy production [7,8]. Unlike carbon dioxide, NO₂ has a relatively short atmospheric lifetime of only several hours [9] and typically remains close to its emission source [10]. Thus, satellites can detect NO₂ concentrations with high spatial and temporal precision. These properties make NO₂ a sensitive and timely proxy for tracking changes in economic activity.

A recent study [7] suggests that satellite-derived NO₂ measurements may outperform traditional nighttime lights data in capturing national-level economic fluctuations. NO₂ data offer several advantages: satellites collect observations during daytime hours, when economic activity typically peaks, and they are less affected by poor atmospheric visibility, conflict-related disruptions, or data manipulation. However, existing research has primarily focused on national-level comparisons and has not examined how NO₂ concentrations relate to economic output within specific sectors. In contrast, this is the first study to systematically test sector-level nitrogen dioxide elasticities at the subnational scale in Japan.

We chose Japan as the study location for several reasons. First, Japan’s democratic system helps ensure reliable economic data—an important consideration, as authoritarian governments often report inflated growth figures. Research shows [11] that such governments exaggerate GDP growth by 0.5 to 1.5 percentage points in official reports submitted to organizations such as the World Bank. Nighttime lights data further confirm that autocracies overstate annual GDP growth by about 35 percent [12]. Second, from 2013 to 2023, Japan has been consistently ranked in the 80th-90th percentile across key governance metrics [13]. Transparency International’s 2024 Corruption Perceptions Index scored Japan 71/100 (20th globally) [14], confirming its reliable data reporting for testing satellite-based measures.

Third, the Japanese government provides extensive economic data. In addition to national GDP, it publishes statistics for each prefecture and reports sector-level output within those prefectures. Each prefecture compiles its own accounts using the Prefectural Accounts Standard Methods. The availability of high-resolution satellite-based NO₂ data further strengthens this analysis.

Our paper makes three main contributions. First, whereas most previous studies analyze economic activity at the global or national level, we examine subnational economic activity using prefecture-level data from Japan. Second, we identify sector-specific relationships between NO₂ concentrations and economic output. Third, we utilize NO₂ data at both coarse (0.25° × 0.25°) and fine (0.1° × 0.1°) spatial resolutions, derived from multiple satellite instruments, including the Ozone Monitoring Instrument (OMI) and the TROPOspheric Monitoring Instrument (TROPOMI).

By comparing NO₂ data with detailed subnational economic indicators in Japan, our analysis shows that NO₂ concentrations can complement—and potentially improve upon—traditional proxies such as nighttime lights data.

## Materials and methods

Our analysis integrates multiple datasets encompassing atmospheric measurements, economic indicators, and proxy variables for human activity. Table 1 summarizes the primary data sources used in the study, along with their spatial and temporal characteristics. These datasets offer complementary insights into nitrogen dioxide concentrations and economic activity across Japan, allowing for a multiscale assessment of their relationship over time.

**Table 1:** Summary of used dataset.

Dataset Name	Spatial Resolution	Temporal Availability	Period Used	Source	Application
OMNO2d (OMI)	27.75 kilometers by 27.75 kilometers	Daily	2005-2018	NASA GES DISC [15]	Daily NO₂ column density
OMNO2d High Resolution	11 kilometers by 11 kilometers	Monthly	2005-2018	NASA GES DISC [16]	Monthly NO₂ column density
Ground-level NO₂	1 km	Annually	2005-2018	ACAG, Washington Univ. [17]	Annual surface NO₂ concentrations
TROPOMI NO₂ (Sentinel-5P)	11 kilometers by 11 kilometers	Monthly	2019-2024	ESA/NASA HAQAST [18]	Annual NO₂ column density
DMSP Nighttime Lights	Variable	Annually	2005-2018	NOAA DMSP-OLS [19]	Economic activity proxy
Gross Prefectural Product	Prefecture-level	Annually	2005-2018	[20]	Total and sectoral economic output
Monthly Economic Data	Prefecture-level	Monthly	2019-2024	[21]	High-frequency economic indicators

We used the R statistical software (version 4.4.1) [22] to extract NO₂ and nighttime lights values. Specifically, we used a combination of R packages: sf for spatial vector operations, terra for raster processing, rhdf5 for handling HDF5-formatted satellite data, exactextractr for zonal statistics, and dplyr and data.table for data manipulation. Once the data is extracted, we use Stata for our econometric analysis. All codes are available on the public depository [23].

We implemented systematic quality control procedures to ensure the reliability of satellite-derived nitrogen dioxide measurements. Fill values below −9990 were treated as missing and replaced with NA to exclude invalid observations.

To predict economic activity from NO₂ concentrations, we follow the methodology developed by Henderson et al. (2012) [1]. Let ln(GDP) denote the observed logarithm of gross domestic product and y represent the true (unobserved) level of economic activity. We assume that


$$\begin{array}{c} \text{Ln}(\text{GDP})=\text{y}+{\text{e}}_{\text{g}} \end{array}$$ (1)

where $e_{g}$ is the measurement error of GDP. Similarly, we assume that the logarithm of measured NO₂ concentration is a function of the real economic activity.


$$\begin{array}{c} \text{Ln}\left(\text{N}{\text{O}}_{\text{2}}\right)=\gamma \text{y}+{\text{e}}_{\text{n}} \end{array}$$ (2)

where $e_{n}$ represents the error term. Our goal is to predict Ln(GDP) using information on ln(NO2). Solving equation (2) for y and substituting into equation (1) yields:


$$\begin{array}{c} \text{Ln}(\text{GDP})=\frac{\text{1}}{\gamma }\{\text{Ln}\left(\text{N}{\text{O}}_{\text{2}}\right)-{\text{e}}_{\text{n}}\}+{\text{e}}_{\text{g}} \end{array}$$ (3)

Applying OLS to the above equation will give us a biased estimate of $\frac{1}{\gamma }$ since the error term in equation (3) is correlated with $\text{Ln}\left(\text{N}{\text{O}}_{\text{2}}\right)$ by definition. However, as long as $cov\left(e_{g},e_{n}\right)$=0, the predicted Ln(GDP) obtained by applying OLS to equation (3) will give the best predictor of Ln(GDP) [1]. The assumption of $cov\left(e_{g},e_{n}\right)$=0 means that there is no cofounding factor that affect both Ln(No2) and the measurement of GDP. To minimize potential confounding, we employ panel data with prefecture fixed effects and year fixed effects. These controls account for time-invariant differences in industrial structure across prefectures and common shocks across years that may influence both NO₂ emissions and the measurement of GDP. The equation we estimate is as follows:


$$ln\left({\text{GDP}}_{it}\right)=\beta_{\text{0}}+\beta_{\text{1}}ln\left({{\text{NO}}_{\text{2}}}_{it}\right)+\alpha_{i}+\gamma_{t}+\varepsilon_{it}$$ (4)

The equation (4) tests whether changes in NO₂ concentrations explain differences in economic activity across space and time. The coefficient β₁ represents the expected percentage change in gross prefectural product resulting from a 1 percent change in NO₂ concentration, holding other factors constant. This natural log–log specification allows for a direct interpretation of β₁ as an elasticity: a 1 percent change in NO₂ concentration corresponds to an estimated β₁ percent change in gross prefectural product.

We offer two remarks on equation (4). First, we assume that real economic activity, y, influences NO₂, making NO₂ an endogenous variable. Nevertheless, as shown by Henderson et al. [1], if there are no confounding factors affecting both NO₂ and GDP, predictions based on NO₂ serve as the best predictor. To reduce the possibility of confounding, we include year fixed effects and country fixed effects. These fixed effects control for time trends and country-specific characteristics, such as industrial structure, that may influence both GDP and NO₂ emissions. However, if unobserved factors exist that affect both ln(GDP) and ln(NO₂) but are not captured by the fixed effects, the predicted GDP may be biased, reflecting the influence of such confounding factors.

Second, this study does not aim to estimate real economic activity, y, directly from observed NO₂ concentrations. To estimate the relationship between real economic activity, y, and observed NO₂, one would need to apply an instrumental variable (IV) estimation method, as done in [7], where nighttime lights were used as an instrument for NO₂. Implementing such an IV approach and estimating real economic activity from observed NO₂ is beyond the scope of this paper.

## Results

### Prefecture-level estimates using satellite-derived nitrogen dioxide measurements at a 0.25-degree by 0.25-degree spatial resolution

Table 2 presents summary statistics for the main variables used in the analysis, including both economic and satellite-based indicators.

**Table 2:** Summary statistics for economic and satellite-derived variables.

Variable	Mean	SD	Min	Max
	(1)	(2)	(3)	(4)
Year	2011.5	4.034	2005	2018
Prefecture code	24	13.575	1	47
Ln of gross prefectural product in trill. yen	1.967	0.844	0.548	4.673
Ln of gross prefectural product in agriculture, forestry and fisheries sector in trill. yen	−2.399	0.666	−3.929	−0.139
Ln of gross prefectural product in construction sector in trill. yen	−0.898	0.760	−2.279	1.582
Ln of gross prefectural product in electricity, gas and water sector in trill. yen	−1.584	0.800	−3.142	0.477
Ln of gross prefectural product in manufacturing sector in trill. yen	0.349	0.973	−1.884	2.764
Ln of gross prefectural product in mining sector in trill. yen	−5.107	0.946	−8.217	−2.267
Tropospheric nitrogen dioxide (10¹⁵ molec/cm²; 0.25-degree by 0.25-degree resolution; source: OMI)	1.596	0.750	0.475	4.734
Ln of tropospheric nitrogen dioxide (10¹⁵ molec/cm²; 0.25-degree by 0.25-degree resolution; source: OMI)	0.366	0.450	−0.744	1.555
Weighted tropospheric nitrogen dioxide (10¹⁵ molec/cm²; 0.25-degree by 0.25-degree resolution; source: OMI)	1.597	0.755	0.469	4.689
Ln of weighted tropospheric nitrogen dioxide (10¹⁵ molec/cm²; 0.25-degree by 0.25-degree resolution; source: OMI)	0.364	0.455	−0.756	1.545
Tropospheric nitrogen dioxide (10¹⁵ molec/cm²; 0.1-degree by 0.1-degree resolution; source: OMI)	4.278	2.660	0.781	15.084
Ln of tropospheric nitrogen dioxide (10¹⁵ molec/cm²; 0.1-degree by 0.1-degree resolution; source: OMI)	1.273	0.611	−0.247	2.714
Observations	658			
Ground-level nitrogen dioxide (parts per billion by volume; 0.1-degree by 0.1-degree resolution; source: HAQAST TROPOMI)	0.230	0.132	0.077	0.884
Ln of ground-level nitrogen dioxide (parts per billion by volume, resolution; 0.1-degree by 0.1-degree resolution; source: HAQAST TROPOMI)	−1.581	0.442	−2.568	−0.124
Observations	644			
Year	2021.5	1.711	2019	2024
Prefecture code	24	13.589	1	47
Ln of gross prefectural product in trill. yen	4.477	0.856	3.034	7.178
Tropospheric nitrogen dioxide (10¹⁵ molec/cm²; 0.1-degree by 0.1-degree resolution; source: TROPOMI)	2.226	1.301	0.740	7.149
Ln of tropospheric nitrogen dioxide (10¹⁵ molec/cm²; 0.1-degree by 0.1-degree resolution; source: TROPOMI)	0.670	0.486	−0.302	1.967
Observations	282			

Table 3 presents the results of fixed-effects panel regressions examining the relationship between satellite-derived nighttime lights luminosity, NO₂ concentrations, and prefecture-level gross domestic product in Japan. The analysis compares the explanatory power of these satellite-derived indicators to evaluate their effectiveness as proxies for subnational economic activity.

**Table 3:** Association between nitrogen dioxide concentrations and prefecture-level gross domestic product using 0.25-degree by 0.25-degree spatial resolution data.

Dependent variable	Ln of gross prefectural product in trill. yen				
Estimation model	Fixed effects panel regression				
Variables	(1)	(2)	(3)	(4)	(5)
Ln of nighttime luminosity	0.03257 (0.026)				
Tropospheric nitrogen dioxide		0.02465* (0.013)			
Ln of tropospheric nitrogen dioxide			0.04560* (0.023)		
Weighted tropospheric nitrogen dioxide				0.02385* (0.014)	
Ln of weighted tropospheric nitrogen dioxide					0.04401* (0.024)
Number of prefectures	47	47	47	47	47
Observations	658	658	658	658	658
Years used	2005-2018	2005-2018	2005-2018	2005-2018	2005-2018
(Within country) R-squared	0.665	0.668	0.668	0.667	0.667
Control variables					
Prefecture fixed effects	Yes	Yes	Yes	Yes	Yes
Year fixed effects	Yes	Yes	Yes	Yes	Yes

Nitrogen dioxide concentrations are measured in units of 10¹⁵ molecules per square centimeter and are derived from data with 0.25-degree by 0.25-degree spatial resolution from the Ozone Monitoring Instrument. Weighted tropospheric nitrogen dioxide refers to measurements adjusted using data quality indicators. Clustered robust standard errors, which account for within-prefecture correlation over time, are reported in parentheses. ***p < 0.01, **p < 0.05, *p < 0.1.

Column (1) of Table 3 shows that the natural logarithm of nighttime lights luminosity has a positive but statistically insignificant coefficient (0.03257). This result supports concerns that night-time light data may lack sensitivity in high-income areas, where brightness levels often saturate. Column (2) shows that tropospheric NO₂ concentrations have a positive and statistically significant association with prefectural gross domestic product, with a coefficient of 0.02465, significant at the 10 percent level. Column (3) shows that the natural logarithm of tropospheric NO₂ also has a positive and statistically significant coefficient of 0.04560 (p < 0.1). Thus, a 1 percent increase in NO₂ concentrations corresponds to an estimated 0.046 percent increase in gross prefectural product.

Column (4) presents results using a quality-weighted tropospheric NO₂ variable, which remains positive and statistically significant (0.02385, p < 0.1). Column (5) shows a similar positive and statistically significant coefficient using the natural logarithm of quality-weighted NO₂ (0.04041, p < 0.1). Thus, a 1 percent increase in quality-weighted NO₂ concentration is associated with an estimated 0.040 percent increase in gross prefectural product.

**Fig 1:**
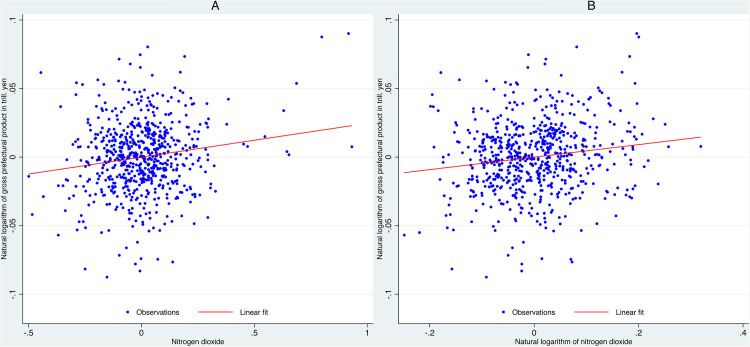
Partial correlation between tropospheric nitrogen dioxide and gross prefectural product after controlling for fixed effects. **(A)** Partial correlation between gross prefectural product and scaled nitrogen dioxide concentrations, controlling for time and prefecture fixed effects. **(B)** Partial correlation between gross prefectural product and the natural logarithm of nitrogen dioxide concentrations, controlling for time and prefecture fixed effects. To generate these figures, we first regress gross prefectural product on time and prefecture fixed effects and plot the resulting residuals on the vertical axis. Similarly, we regress the scaled nitrogen dioxide concentration (or its natural logarithm for Figure B) on the same fixed effects and plot the residuals on the horizontal axis.

Fig 1 shows the partial correlation between NO₂ concentrations and gross prefectural product after controlling for prefecture fixed effects and year fixed effects. We regress NO₂ concentrations on prefecture and year fixed effects and calculate the residuals. Similarly, we regress gross prefectural product on the same fixed effects and calculate the residuals. We then plot the NO₂ residuals on the horizontal axis and the gross prefectural product residuals on the vertical axis.

These residual plots depict the partial correlation between economic activity and NO₂ concentrations, removing variation due to time-invariant prefecture characteristics and year-specific shocks. The fitted red lines display a slight upward slope, indicating a positive association. The slope coefficients correspond to those estimated in the full fixed-effects panel regressions.

**Fig 2:**
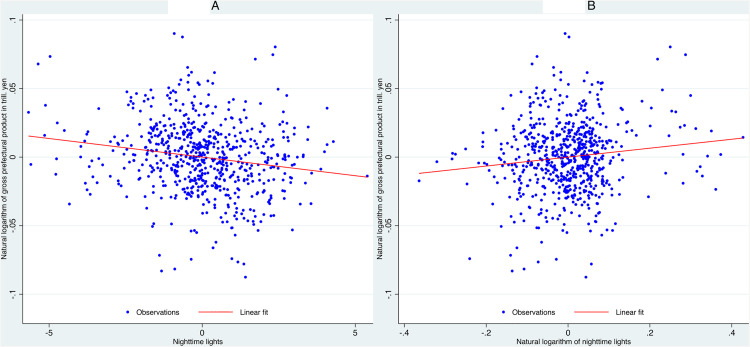
Partial correlation between nighttime light luminosity and gross prefectural product after controlling for fixed effects. (A) Partial correlation between gross prefectural product and nighttime lights luminosity, controlling for time and prefecture fixed effects. (B) Partial correlation between gross prefectural product and the natural logarithm of nighttime lights luminosity, controlling for time and prefecture fixed effects. The same method used to generate Fig 1 is applied here.

We apply the same residual analysis to nighttime lights luminosity and gross prefectural product, controlling for unobserved heterogeneity through fixed effects (Fig 2).

These residual plots depict the conditional relationship between economic activity and nighttime lights intensity, removing variation due to time-invariant prefecture characteristics and year-specific shocks. Panel (A) shows a negative association, with a downward-sloping fitted line and wide dispersion of observations. Panel (B) displays a modest positive association, with an upward-sloping fitted line.

Thus, Fig 1 demonstrates that NO₂ maintains a consistently positive relationship with gross prefectural product, whether measured in raw terms or as natural logarithms. This suggests that the relationship between NO₂ and gross prefectural product is more stable and approximately linear, with both absolute and percentage changes in NO₂ closely tracking economic activity.

We also analyze sector-specific outcomes to identify which industries are most effectively captured by satellite-based indicators and to evaluate whether NO₂ concentrations or nighttime lights luminosity more accurately reflect economic activity within each sector.

Table 4 presents the results of fixed-effects panel regressions examining the relationship between satellite-derived nighttime lights luminosity, NO₂ concentrations, and gross prefectural product in Japan’s mining sector at the prefectural level.

**Table 4:** Association between nitrogen dioxide concentrations and prefecture-level gross domestic product in the mining sector using 0.25-degree by 0.25-degree spatial resolution data.

Dependent variable	Ln of gross prefectural product in mining sector in trill. yen				
Estimation model	Fixed effects panel regression				
Variables	(1)	(2)	(3)	(4)	(5)
Ln of nighttime luminosity	0.52093** (0.219)				
Tropospheric nitrogen dioxide		0.30893*** (0.092)			
Ln of tropospheric nitrogen dioxide			0.61428*** (0.190)		
Weighted tropospheric nitrogen dioxide				0.31626*** (0.096)	
Ln of weighted tropospheric nitrogen dioxide					0.63762*** (0.203)
Number of prefectures	47	47	47	47	47
Observations	658	658	658	658	658
Years used	2005-2018	2005-2018	2005-2018	2005-2018	2005-2018
(Within country) R-squared	0.244	0.245	0.248	0.245	0.248
Control variables					
Prefecture fixed effects	Yes	Yes	Yes	Yes	Yes
Year fixed effects	Yes	Yes	Yes	Yes	Yes

Nitrogen dioxide concentrations are measured in units of 10¹⁵ molecules per square centimeter and are derived from data with 0.25-degree by 0.25-degree spatial resolution from the Ozone Monitoring Instrument. Weighted tropospheric nitrogen dioxide refers to measurements adjusted using data quality indicators. Clustered robust standard errors, accounting for within-prefecture correlation over time, are reported in parentheses. ***p < 0.01, **p < 0.05, *p < 0.1.

Column (1) of Table 4 shows that the natural logarithm of nighttime lights luminosity has a positive and statistically significant coefficient (0.52093), significant at the 5 percent level. This result suggests that nighttime lights luminosity may still serve as a relevant proxy for mining activity. Column (2) shows that tropospheric NO₂ concentrations have a positive and statistically significant association with prefectural mining gross domestic product, with a coefficient of 0.30893, significant at the 1 percent level. Column (3) shows that the natural logarithm of tropospheric NO₂ also has a positive and statistically significant coefficient of 0.61422 (p < 0.01), indicating that a 1 percent increase in NO₂ concentrations corresponds to an estimated 0.614 percent increase in mining GDP. Column (4) presents results using a quality-weighted tropospheric NO₂ variable, which also yields a positive and statistically significant coefficient (0.31626, p < 0.01). Column (5) shows a similar positive and statistically significant coefficient using the natural logarithm of quality-weighted NO₂ (0.63762, p < 0.01). Thus, a 1 percent increase in quality-weighted NO₂ concentrations is associated with an estimated 0.638 percent increase in mining gross domestic product.

The results in Table 4 reveal a consistent and statistically significant relationship between tropospheric NO₂ concentrations and gross prefectural product in the mining sector across all model specifications. Although both nighttime lights luminosity and NO₂ concentrations are positively associated with economic output, NO₂ shows stronger correlations and greater statistical significance. This likely stems from the direct connection between NO₂ emissions and the combustion processes involved in mining. As a result, NO₂ provides a more reliable proxy for monitoring mining sector dynamics, supporting near real-time production tracking and leveraging the advantages of global satellite coverage.

The NO₂ elasticity in the mining sector (0.61428) is much higher than that in gross prefectural product (0.04560). At first glance, such a large elasticity may appear implausible. However, this result is reasonable for two main reasons. First, the mining sector accounts for only about 1.25% of Japan’s total GDP [24]. A high elasticity within this small sector is therefore consistent with the low elasticity of total prefectural output. Second, the main sub-industries—quarrying (42.6%), crude oil and gas extraction (24.6%), and ceramic minerals (21.0%)—are all highly emission-intensive. Quarrying and oil and gas extraction emit large amounts of NO₂ from diesel equipment, explosives, and gas flaring. The ceramic minerals industry also releases NO₂ from diesel machinery, blasting, and kiln firing. These processes generate substantial emissions relative to output, explaining why the mining sector exhibits a high NO₂ elasticity.

Table 5 presents the results of fixed-effects panel regressions analyzing the relationship between satellite-derived nighttime lights luminosity, NO₂ concentrations, and gross prefectural product in Japan’s electricity, gas, and water utilities sector.

**Table 5:** Association between nitrogen dioxide concentrations and prefecture-level gross domestic product in the electricity, gas and water sector using 0.25-degree by 0.25-degree spatial resolution data.

Dependent variable	Ln of gross prefectural product in electricity, gas and water sector in trill. yen				
Estimation model	Fixed effects panel regression				
Variables	(1)	(2)	(3)	(4)	(5)
Ln of nighttime luminosity	0.25622** (0.126)				
Tropospheric nitrogen dioxide		0.10579* (0.053)			
Ln of tropospheric nitrogen dioxide			0.25636** (0.123)		
Weighted tropospheric nitrogen dioxide				0.10651* (0.057)	
Ln of weighted tropospheric nitrogen dioxide					0.24456* (0.132)
Number of prefectures	47	47	47	47	47
Observations	658	658	658	658	658
Years used	2005-2018	2005-2018	2005-2018	2005-2018	2005-2018
(Within country) R-squared	0.471	0.464	0.469	0.464	0.467
Control variables					
Prefecture fixed effects	Yes	Yes	Yes	Yes	Yes
Year fixed effects	Yes	Yes	Yes	Yes	Yes

Nitrogen dioxide concentrations are measured in units of 10¹⁵ molecules per square centimeter and are derived from data with 0.25-degree by 0.25-degree spatial resolution from the Ozone Monitoring Instrument. Weighted tropospheric nitrogen dioxide refers to measurements adjusted using data quality indicators. Clustered robust standard errors, reported in parentheses, account for within-prefecture correlation over time. ***p < 0.01, **p < 0.05, *p < 0.1.

Column (1) of Table 5 shows that the natural logarithm of nighttime lights luminosity has a positive and statistically significant coefficient of 0.25622, significant at the 5 percent level. This result suggests that nighttime lights effectively capture activity in the electricity, gas, and water sector, likely reflecting the sector’s reliance on infrastructure and consistent energy usage. Column (2) shows that tropospheric NO₂ concentrations are positively and statistically significantly associated with sectoral gross domestic product, with a coefficient of 0.10579, significant at the 10 percent level. Column (3) presents results using the natural logarithm of tropospheric NO₂, which has a positive and statistically significant coefficient of 0.25636 (p < 0.05), implying that a 1 percent increase in NO₂ concentrations is associated with an estimated 0.256 percent increase in output in the electricity, gas, and water sector. Column (4) uses a quality-weighted tropospheric NO₂ variable and yields a positive and statistically significant coefficient of 0.10651 at the 10 percent level. Column (5) shows that the natural logarithm of quality-weighted NO₂ remains positively and statistically significantly associated with sectoral gross prefectural product, with a coefficient of 0.24456 (p < 0.1), indicating that a 1 percent increase in quality-weighted NO₂ concentrations corresponds to an approximate 0.245 percent increase in economic output for this sector.

The results in Table 5 indicate that both nighttime lights luminosity and tropospheric NO₂ concentrations serve as relevant proxies for estimating economic activity in the electricity, gas, and water sector. Although the nighttime lights luminosity coefficient is statistically significant and positive, suggesting some usefulness in capturing infrastructure-driven activity, the NO₂ indicators consistently exhibit stronger coefficients and greater statistical significance across specifications. This likely reflects the direct emissions of nitrogen oxides from thermal power plants, which dominate Japan’s electricity generation and emit substantial quantities of NO₂ during combustion. As a result, NO₂ proves to be particularly effective for near real-time monitoring of changes in this sector.

Table 6 presents fixed-effects panel regressions results examining the relationship between satellite-derived nighttime lights luminosity, NO₂ concentrations, and gross prefectural product in Japan’s agriculture, forestry, and fisheries sector.

**Table 6:** Association between nitrogen dioxide concentrations and prefecture-level gross domestic product in the agriculture, forestry, and fisheries sector using 0.25-degree by 0.25-degree spatial resolution data.

Dependent variable	Ln of gross prefectural product in agriculture, forestry and fisheries sector in trill. yen				
Estimation model	Fixed effects panel regression				
Variables	(1)	(2)	(3)	(4)	(5)
Ln of nighttime luminosity	−0.10612 (0.133)				
Tropospheric nitrogen dioxide		−0.04544* (0.025)			
Ln of tropospheric nitrogen dioxide			−0.09149 (0.060)		
Weighted tropospheric nitrogen dioxide				−0.04692* (0.024)	
Ln of weighted tropospheric nitrogen dioxide					−0.09773* (0.058)
Number of prefectures	47	47	47	47	47
Observations	658	658	658	658	658
Years used	2005-2018	2005-2018	2005-2018	2005-2018	2005-2018
(Within country) R-squared	0.312	0.306	0.307	0.306	0.307
Control variables					
Prefecture fixed effects	Yes	Yes	Yes	Yes	Yes
Year fixed effects	Yes	Yes	Yes	Yes	Yes

Nitrogen dioxide concentrations are measured in units of 10¹⁵ molecules per square centimeter and are derived from data with 0.25-degree by 0.25-degree spatial resolution from the Ozone Monitoring Instrument. Weighted tropospheric nitrogen dioxide refers to measurements adjusted using data quality indicators. Clustered robust standard errors, reported in parentheses, account for within-prefecture correlation over time. ***p < 0.01, **p < 0.05, *p < 0.1.

Column (1) of Table 6 shows that the natural logarithm of nighttime lights luminosity has a negative but statistically insignificant coefficient of –0.10612. This result suggests that nighttime lights are poorly suited to capturing activity in the agriculture, forestry, and fisheries sector, likely due to the sector’s rural nature and low luminosity levels. Column (2) shows that tropospheric NO₂ concentrations are negatively and statistically significantly associated with sectoral gross domestic product, with a coefficient of –0.04514, significant at the 10 percent level. Column (3) presents results using the natural logarithm of tropospheric NO₂, which yields a negative but statistically insignificant coefficient of –0.09149, indicating a weaker and less precise relationship. Column (4) uses a quality-weighted tropospheric NO₂ variable and produces a negative and statistically significant coefficient of –0.04692 at the 10 percent level. Column (5) shows that the natural logarithm of quality-weighted NO₂ remains negatively and statistically significantly associated with sectoral gross prefectural product, with a coefficient of –0.09773 (p < 0.1), indicating that a 1 percent increase in quality-weighted NO₂ concentrations corresponds to an approximate 0.098 percent decline in economic output for this sector.

Forests play a significant role in mitigating air pollution by acting as natural sinks for NO₂, which may account for the negative association observed in this sector. Through dry deposition, forests absorb NO₂ onto leaf surfaces, where it is stored, transformed, or assimilated into plant tissues. For example, trees across the United States removed an estimated 711,000 metric tons of air pollutants, including substantial amounts of NO₂, in 1994 [25]. To examine this mechanism, we conducted regressions that incorporated forest cover for each prefecture and year (S1 Table A). Once forest area was included, the absolute value of the NO₂ coefficient declined by nearly half and became statistically insignificant. When both forest area and population size were included (S1 Table B), the results remained largely consistent with those in S1 Table A, indicating that forest cover plays a dominant role, while population size has only a limited influence in explaining the results in Table 6. Overall, these findings suggest that forest cover largely explains the negative correlation between NO₂ concentrations and gross prefectural product in agriculture-related sectors, supporting the hypothesis that forests help absorb NO₂.

Table 7 presents the results of fixed-effects panel regressions analyzing the relationship between satellite-derived nighttime lights luminosity, NO₂ concentrations, and gross prefectural product in Japan’s construction sector.

**Table 7:** Association between nitrogen dioxide concentrations and prefecture-level gross domestic product in the construction sector using 0.25-degree by 0.25-degree spatial resolution data.

Dependent variable	Ln of gross prefectural product in construction sector in trill. yen				
Estimation model	Fixed effects panel regression				
Variables	(1)	(2)	(3)	(4)	(5)
Ln of nighttime luminosity	0.03571 (0.081)				
Tropospheric nitrogen dioxide		0.06153* (0.035)			
Ln of tropospheric nitrogen dioxide			0.13308 (0.090)		
Weighted tropospheric nitrogen dioxide				0.06572* (0.037)	
Ln of weighted tropospheric nitrogen dioxide					0.13863 (0.097)
Number of prefectures	47	47	47	47	47
Observations	658	658	658	658	658
Years used	2005-2018	2005-2018	2005-2018	2005-2018	2005-2018
(Within country) R-squared	0.287	0.292	0.293	0.292	0.293
Control variables					
Prefecture fixed effects	Yes	Yes	Yes	Yes	Yes
Year fixed effects	Yes	Yes	Yes	Yes	Yes

Nitrogen dioxide concentrations are measured in units of 10¹⁵ molecules per square centimeter and are derived from data with 0.25-degree by 0.25-degree spatial resolution from the Ozone Monitoring Instrument. Weighted tropospheric nitrogen dioxide refers to measurements adjusted using data quality indicators. Clustered robust standard errors, reported in parentheses, account for within-prefecture correlation over time. ***p < 0.01, **p < 0.05, *p < 0.1.

Column (1) of Table 7 shows that the natural logarithm of nighttime lights luminosity has a positive but statistically insignificant coefficient of 0.03571. This result indicates the limited explanatory power of nighttime lights in capturing economic activity in the construction sector, potentially due to the sector’s irregular lighting patterns and the temporal concentration of work. Column (2) shows that tropospheric NO₂ concentrations are positively and statistically significantly associated with construction gross domestic product, with a coefficient of 0.06153, significant at the 10 percent level. Column (3) uses the natural logarithm of tropospheric NO₂ and yields a positive but statistically insignificant coefficient of 0.13308, indicating a weaker and less precise relationship. Column (4) uses a quality-weighted tropospheric NO₂ variable and produces a positive and statistically significant coefficient of 0.06572 at the 5 percent level, reinforcing the relationship between atmospheric NO₂ concentrations and construction activity when controlling for data quality. Column (5) shows that the natural logarithm of quality-weighted NO₂ remains positively but statistically insignificantly associated with sectoral gross prefectural product, with a coefficient of 0.13863.

Overall, the results suggest a positive association between NO₂ concentrations and construction-sector gross prefectural product.

Table 8 presents the results of fixed-effects panel regressions analyzing the relationship between satellite-derived nighttime lights luminosity, NO₂ concentrations, and gross prefectural product in Japan’s manufacturing sector.

Column (1) of Table 8 shows that the natural logarithm of nighttime lights has a negative but statistically insignificant coefficient (–0.06681). Column (2) shows that tropospheric NO₂ concentrations have a positive coefficient of 0.06164, indicating a direct relationship between NO₂ concentrations and manufacturing gross prefectural product. Column (3) presents results using the natural logarithm of tropospheric NO₂ concentrations, with a coefficient of 0.05796. Thus, a 1% increase in NO₂ concentrations is associated with a 0.058% increase in manufacturing gross prefectural product. Column (4) uses weighted tropospheric NO₂ concentrations and yields a positive coefficient of 0.05403. Column (5) shows results using the natural logarithm of weighted tropospheric NO₂ concentrations, with a positive coefficient of 0.04511. This suggests that a 1% increase in quality-weighted NO₂ concentrations corresponds to a 0.045% increase in manufacturing gross prefectural product.

**Table 8:** Association between nitrogen dioxide concentrations and prefecture-level gross domestic product in the manufacturing sector using 0.25-degree by 0.25-degree spatial resolution data.

Dependent variable	Ln of gross prefectural product in manufacturing sector in trill. yen				
Estimation model	Fixed effects panel regression				
Variables	(1)	(2)	(3)	(4)	(5)
Ln of nighttime luminosity	−0.06681 (0.045)				
Tropospheric nitrogen dioxide		0.06164 (0.057)			
Ln of tropospheric nitrogen dioxide			0.05796 (0.081)		
Weighted tropospheric nitrogen dioxide				0.05403 (0.061)	
Ln of weighted tropospheric nitrogen dioxide					0.04511 (0.084)
Number of prefectures	47	47	47	47	47
Observations	658	658	658	658	658
Years used	2005-2018	2005-2018	2005-2018	2005-2018	2005-2018
(Within country) R-squared	0.593	0.597	0.592	0.595	0.591
Control variables					
Prefecture fixed effects	Yes	Yes	Yes	Yes	Yes
Year fixed effects	Yes	Yes	Yes	Yes	Yes

Nitrogen dioxide concentrations are measured in units of 10¹⁵ molecules per square centimeter and are derived from data with 0.25-degree by 0.25-degree spatial resolution from the Ozone Monitoring Instrument. Weighted tropospheric nitrogen dioxide refers to measurements adjusted using data quality indicators. Clustered robust standard errors, reported in parentheses, account for within-prefecture correlation over time. ***p < 0.01, **p < 0.05, *p < 0.1.

The results in Table 8 show consistently positive coefficients across all NO₂ specifications, suggesting a directional association between NO₂ concentrations and output in the manufacturing sector. Although these estimates lack statistical significance, the magnitude and consistency of the coefficients imply that NO₂ concentrations may serve as a useful proxy for industrial activity in emissions-intensive sectors such as manufacturing.

One potential concern in the above analysis is the presence of confounding factors that influence both measured economic activity and observed NO₂ concentrations. Weather conditions represent one such possible factor. S1 Fig. A, S1 Fig. B, S1 Fig. C, S1 Fig. D S1 Fig. E, S1 Fig. F present the estimated coefficients of each NO₂-related variable, both with and without weather-related controls (annual rainfall and temperature). The results show that the estimated coefficients of the NO₂-related variables remain nearly identical across specifications, suggesting that weather conditions are unlikely to be a significant confounding factor.

The sectoral analysis reveals variation in the performance of satellite-derived indicators across economic sectors. NO₂ concentrations show exceptionally strong associations with the mining, utilities, construction, and manufacturing sectors, where output is closely linked to direct emissions. In contrast, the agriculture, forestry, and fisheries sector exhibit negative associations with NO₂, likely reflecting the role of land-based activities as environmental sinks for air pollutants.

### Does the use of higher-resolution data generate better results? Analysis using 0.1-degree by 0.1-degree spatial resolution nitrogen dioxide data

Table 9 shows the results of fixed-effects panel regressions examining the relationship between satellite-derived nitrogen dioxide concentrations and prefecture-level gross domestic product in Japan, using 0.1-degree by 0.1-degree spatial resolution data from multiple data sources.

**Table 9:** Association between nitrogen dioxide concentrations and prefecture-level gross domestic product using 0.1-degree by 0.1-degree spatial resolution data.

Dependent variable	Ln of gross prefectural product in trill. yen					
Estimation model	Fixed effects panel regression					
Variables	(1)	(2)	(3)	(4)	(5)	(6)
Tropospheric nitrogen dioxide (source: OMI)	−0.00830** (0.004)					
Ln of tropospheric nitrogen dioxide (source: OMI)		−0.03836 (0.034)				
Ground-level nitrogen dioxide (source: HAQAST TROPOMI)			0.00669 (0.028)			
Ln of ground-level nitrogen dioxide (source: HAQAST TROPOMI)				−0.00360 (0.012)		
Tropospheric nitrogen dioxide (source: TROPOMI)					−0.00734 (0.006)	
Ln of tropospheric nitrogen dioxide (source: TROPOMI)						−0.03475 (0.025)
Number of prefectures	47	47	46	46	47	47
Observations	658	658	644	644	282	282
Years used	2005-2018	2005-2018	2005-2018	2005-2018	2019-2024	2019-2024
(Within country) R-squared	0.670	0.664	0.673	0.673	0.624	0.625
Control variables						
Prefecture fixed effects	Yes	Yes	Yes	Yes	Yes	Yes
Year fixed effects	Yes	Yes	Yes	Yes	Yes	Yes

Nitrogen dioxide concentrations are measured in units of 10¹⁵ molecules per square centimeter and are derived from satellite products with a spatial resolution of 0.1 degrees by 0.1 degrees. The number of observations varies across columns due to differences in data sources and availability. Columns (1) and (2) use tropospheric nitrogen dioxide data from the OMNO₂d dataset, based on OMI satellite observations provided by NASA, covering 2005–2018 for 47 prefectures (658 observations = 47 prefectures × 14 years). Columns (3) and (4) use ground-level nitrogen dioxide concentrations, expressed in parts per billion by volume, from the HAQAST TROPOMI dataset developed by the Atmospheric Composition Analysis Group at Washington University in St. Louis. Okinawa Prefecture is excluded from these columns due to data unavailability, resulting in 46 prefectures over 2005–2018 (644 observations = 46 prefectures × 14 years). Columns (5) and (6) use tropospheric nitrogen dioxide data from the TROPOMI satellite product, which are only available from 2019 onward, covering 2019–2024 for 47 prefectures (282 observations = 47 prefectures × 6 years). Clustered robust standard errors, reported in parentheses, account for within-prefecture correlation over time. ***p < 0.01, **p < 0.05, *p < 0.1.

Column (1) of Table 9 contrasts with prior findings by showing that tropospheric nitrogen dioxide exhibits a negative and statistically significant association with prefecture-level gross domestic product. The coefficient of –0.00830 is significant at the 1 percent level, indicating an inverse relationship between satellite-derived nitrogen dioxide concentrations at 0.1-degree by 0.1-degree resolution and economic output. Column (2), which uses the natural logarithm of tropospheric nitrogen dioxide, produces a negative but statistically insignificant coefficient of –0.03836.

Column (3) incorporates ground-level nitrogen dioxide measurements and yields a positive, though statistically insignificant, coefficient of 0.00669. Column (4), which uses the logarithmic form of ground-level nitrogen dioxide, also has a negative and statistically insignificant coefficient of –0.00360.

Columns (5) and (6) use tropospheric nitrogen dioxide data from the TROPOMI satellite product, available only from 2019 onward. These specifications include fewer observations than Columns (1) through (4). In addition, Columns (5) and (6) rely on a different source of gross prefectural product than the earlier columns. Column (5) reports a negative but statistically insignificant coefficient of –0.00734. Column (6), which uses the natural logarithm of the same variable, yields a similar result (–0.03475), also statistically insignificant.

While higher-resolution satellite NO₂ data (0.1°) might intuitively seem to improve measurement accuracy, our analysis suggests the opposite. This counterintuitive result can be attributed to three main factors: atmospheric noise, retrieval uncertainty, and spatial mismatch with economic data. First, at smaller grid sizes, satellite instruments such as TROPOMI become more sensitive to transient meteorological conditions (cloud cover, aerosol interference, or wind dispersion), which reduces the signal-to-noise ratio of NO₂ column densities [26]. Second, high-resolution retrievals are more prone to artifacts caused by surface reflectance and algorithmic limitations [27]. While these errors may average out at coarser spatial scales, they become more pronounced at 0.1°, introducing measurement error into the key explanatory variable. According to classical errors-in-variables theory, this type of error leads to attenuation bias, which may account for both the loss of statistical significance and the inconsistent coefficient signs observed in our high-resolution estimations [28]. Third, economic indicators such as prefecture-level GDP can be spatially aggregated and inherently mismatched with high-resolution NO₂ grids, especially in areas with heterogeneous land use or emission intensity. Using 0.25° data provides a natural averaging effect that better aligns with administrative boundaries and suppresses the influence of localized outliers, such as highways or small industrial zones [29]. The modifiable areal unit problem (MAUP) further complicates inference: changing the spatial resolution of explanatory variables can significantly alter estimated relationships [30]. Higher-resolution data tend to capture more localized variability and idiosyncratic noise, which may not correspond to the aggregated nature of economic output. As a result, increasing spatial resolution can weaken the true signal and distort coefficients [31]. These findings underscore the importance of scale compatibility in satellite-based economic measurement and support the use of moderately aggregated NO₂ data (0.25°) for robust inference.

## Discussion

We evaluated satellite-derived tropospheric NO₂ concentrations as a proxy for measuring subnational economic activity in Japan, a country recognized for its transparent and high-quality economic statistics.

Unlike nighttime lights data, which often saturate in urban areas and fail to capture daytime activity, NO₂ concentrations provide higher temporal sensitivity due to their short atmospheric lifetime and direct links to combustion-related sectors. This makes nitrogen dioxide a potentially valuable proxy for monitoring economic activity in sectors such as transportation, power generation, and industrial manufacturing, where emissions are closely tied to combustion processes.

The analysis demonstrates significant positive elasticities between NO₂ concentrations and gross prefectural product at a 0.25° spatial resolution—particularly in sectors with intensive fossil fuel use, such as mining (elasticity = 0.63), utilities (elasticity = 0.25), and construction (elasticity = 0.13). In contrast, the agriculture, forestry, and fisheries sector exhibit a negative elasticity (–0.09), consistent with its pollutant-absorbing function.

A key strength of this analysis is the integration of NO₂ data with reliable subnational economic statistics, enabling robust validation. Moreover, the results reveal substantial differences across economic sectors, demonstrating that NO₂ more accurately reflects output dynamics, particularly in fossil fuel–intensive sectors such as mining, utilities, and manufacturing. Additionally, the findings challenge the common assumption that higher spatial resolution improves economic measurement, showing instead that it may introduce atmospheric noise or temporal mismatches.

These results reinforce and extend earlier research on satellite-based economic indicators, particularly recent work [7] showing that NO₂ concentrations outperform nighttime lights data in capturing national-level economic trends. Although nighttime lights data have proven useful as proxies for economic activity [1], their effectiveness declines in countries with well-developed statistical systems [2]; in densely populated urban areas with limited brightness variation [4]; and in rural regions with low population density and limited electrification [5]. Our paper advances the literature by validating NO₂ as a more temporally responsive and sectorally informative alternative at the subnational level.

Despite its contributions, this study has several limitations. First, the use of annual gross prefectural product data restricts our ability to capture short-term economic fluctuations. Second, NO₂ concentrations are influenced by meteorological conditions and topography, which may introduce variation unrelated to economic activity. Third, omitted confounding factors may exist. Although economic activity affects NO₂ through combustion-related emissions, environmental regulations and pollution control policies can also shape both industrial structure and emission levels, creating potential endogeneity. In such cases, estimating the structural relationship from economic activity to NO₂ emissions using instrumental variable techniques is necessary. Fourth, uncertainties in satellite retrievals may affect measurement accuracy. Persistent cloud cover can reduce data availability, and long-term calibration drift may generate artificial trends unrelated to true changes in NO₂ or economic activity. Fifth, the external validity of the findings for countries with weaker statistical systems, inconsistent satellite coverage, or large informal sectors remains uncertain.

Finally, the current analysis may not be directly applicable to economies with a substantially higher share of service sectors. In Japan, the service sector accounted for 69.3% of GDP in 2018, according to the Cabinet Office’s National Accounts [32]. Despite this high share, we still find a positive and statistically significant relationship between prefectural GDP and NO₂ concentrations. Although speculative, it is plausible that service-sector activities are closely interconnected with industrial production. For instance, when manufacturing firms increase output, their demand for related services—such as financing, logistics, advertising, and business transactions—also rises. This interdependence suggests that, when properly calibrated, NO₂ can serve as a useful indicator of overall economic activity even in high-income countries.

However, nitrogen dioxide concentrations may be less effective in capturing economic activity in countries where the service sector constitutes an even larger proportion of GDP. In economies with a substantial presence of information technology, healthcare, or education industries, the relationship between NO₂ emissions and economic activity may differ considerably. Exploring the GDP–NO₂ relationship in such contexts remains an important avenue for future research.

Given these considerations, this study should be regarded as a benchmark for future investigations rather than a universal model.

Future research could proceed in several directions. First, linking NO₂ data with monthly or quarterly economic indicators could enhance the detection of high-frequency emission dynamics. Second, applying this approach to lower-income countries using microdata with GPS information could help evaluate its applicability in contexts where conventional economic statistics are limited or unreliable.

From a policy perspective, these findings support the use of NO₂ concentrations in economic analysis. This indicator could offer timely, geographically detailed, and sector-sensitive insights—particularly valuable during economic crises and shocks.

In conclusion, this paper demonstrates that tropospheric NO₂ concentrations can serve as a valid and sector-specific proxy for subnational economic activity. The analysis also confirms its potential as a policy-relevant metric and highlights the broader use of satellitebased indicators in analyzing regional economic performance.

## Supporting information

S1 Table Supporting information tables. (DOCX)

S1 Fig Supporting information figures. (DOCX)
