# Supplementary material for: Predicting economic activity using atmospheric nitrogen dioxide (NO2) satellite data: Evidence from local economic indicators in Japan
Source: PLoS One. 2025 Dec 4;20(12):e0337901. doi: 10.1371/journal.pone.0337901 (PMC12677523; doi:10.1371/journal.pone.0337901)
Supplement: S1 Table — (DOCX) [file pone.0337901.s001.docx]

**S1 Table**

**S1 Table A. Association between nitrogen dioxide concentrations and prefecture-level gross domestic product in the agriculture, forestry, and fisheries sector using 0.25-degree by 0.25-degree spatial resolution data with ln of forest area.**

| Dependent Variable | Ln of gross prefectural product in agriculture, forestry and fisheries sector in trill. yen | | | | |
| --- | --- | --- | --- | --- | --- |
| Estimation Model | Fixed Effect Panel Regression | | | | |
| Variables | (1) | (2) | (3) | (4) | (5) |
| Ln of night-time luminosity | -0.05141 |  |  |  |  |
|  | (0.103) |  |  |  |  |
| Tropospheric nitrogen dioxide |  | -0.02119 |  |  |  |
|  |  | (0.021) |  |  |  |
| Ln of tropospheric nitrogen dioxide |  |  | -0.04556 |  |  |
|  |  |  | (0.043) |  |  |
| Weighted tropospheric nitrogen dioxide |  |  |  | -0.02314 |  |
|  |  |  |  | (0.022) |  |
| Ln of weighted tropospheric nitrogen dioxide |  |  |  |  | -0.05366 |
|  |  |  |  |  | (0.043) |
| Ln of forest area, km2 | -3.41771 | -3.43798 | -3.34156 | -3.47123 | -3.38059 |
|  | (2.132) | (2.130) | (2.116) | (2.131) | (2.112) |
| Number of prefectures | 47 | 47 | 47 | 47 | 47 |
| Observations | 517 | 517 | 517 | 517 | 517 |
| Years used | 2005-2015 | 2005-2015 | 2005-2015 | 2005-2015 | 2005-2015 |
| (Within country) R-squared | 0.401 | 0.400 | 0.401 | 0.401 | 0.402 |
| Control variables |  |  |  |  |  |
| Prefecture fixed effects | Yes | Yes | Yes | Yes | Yes |
| Year fixed effects | Yes | Yes | Yes | Yes | Yes |

Nitrogen dioxide concentrations are measured in units of 10¹⁵ molecules per square centimeter and are derived from data with 0.25-degree by 0.25-degree spatial resolution from the Ozone Monitoring Instrument. Weighted tropospheric nitrogen dioxide refers to measurements adjusted using data quality indicators. Clustered robust standard errors, which account for within-prefecture correlation over time, are reported in parentheses. ***p < 0.01, **p < 0.05, *p < 0.1.

**S1 Table B. Association between nitrogen dioxide concentrations and prefecture-level gross domestic product in the agriculture, forestry, and fisheries sector using 0.25-degree by 0.25-degree spatial resolution data with ln of forest and ln of population.**

| Dependent Variable | Ln of gross prefectural product in agriculture, forestry and fisheries sector in trill. yen | | | | |
| --- | --- | --- | --- | --- | --- |
| Estimation Model | Fixed Effect Panel Regression | | | | |
| Variables | (1) | (2) | (3) | (4) | (5) |
| Ln of night-time luminosity | -0.06439 |  |  |  |  |
|  | (0.095) |  |  |  |  |
| Tropospheric nitrogen dioxide |  | -0.02309 |  |  |  |
|  |  | (0.021) |  |  |  |
| Ln of tropospheric nitrogen dioxide |  |  | -0.05137 |  |  |
|  |  |  | (0.042) |  |  |
| Weighted tropospheric nitrogen dioxide |  |  |  | -0.02470 |  |
|  |  |  |  | (0.022) |  |
| Ln of weighted tropospheric nitrogen dioxide |  |  |  |  | -0.05845 |
|  |  |  |  |  | (0.042) |
| Ln of forest area, km2 | -3.22644 | -3.25934 | -3.14701 | -3.29267 | -3.18843 |
|  | (2.198) | (2.187) | (2.160) | (2.188) | (2.160) |
| Ln of population | 0.36872 | 0.30233 | 0.32063 | 0.29809 | 0.31500 |
|  | (0.451) | (0.514) | (0.512) | (0.513) | (0.511) |
| Number of prefectures | 47 | 47 | 47 | 47 | 47 |
| Observations | 517 | 517 | 517 | 517 | 517 |
| Years used | 2005-2015 | 2005-2015 | 2005-2015 | 2005-2015 | 2005-2015 |
| (Within country) R-squared | 0.312 | 0.306 | 0.307 | 0.306 | 0.307 |
| Control variables |  |  |  |  |  |
| Prefecture fixed effects | Yes | Yes | Yes | Yes | Yes |
| Year fixed effects | Yes | Yes | Yes | Yes | Yes |

Nitrogen dioxide concentrations are measured in units of 10¹⁵ molecules per square centimeter and are derived from data with 0.25-degree by 0.25-degree spatial resolution from the Ozone Monitoring Instrument. Weighted tropospheric nitrogen dioxide refers to measurements adjusted using data quality indicators. Clustered robust standard errors, which account for within-prefecture correlation over time, are reported in parentheses. ***p < 0.01, **p < 0.05, *p < 0.1.
