# Supplementary material for: Predicting economic activity using atmospheric nitrogen dioxide (NO2) satellite data: Evidence from local economic indicators in Japan
Source: PLoS One. 2025 Dec 4;20(12):e0337901. doi: 10.1371/journal.pone.0337901 (PMC12677523; doi:10.1371/journal.pone.0337901)
Supplement: S1 Fig — (DOCX) [file pone.0337901.s002.docx]

**S1 Figures. Coefficient plots for baseline and climate-controlled estimates of NO₂ and GDP associations.**

**S1 Fig. A. Baseline and Precipitation- and Temperature-Controlled Estimates: Association between NO₂ and Prefecture GDP.**

**
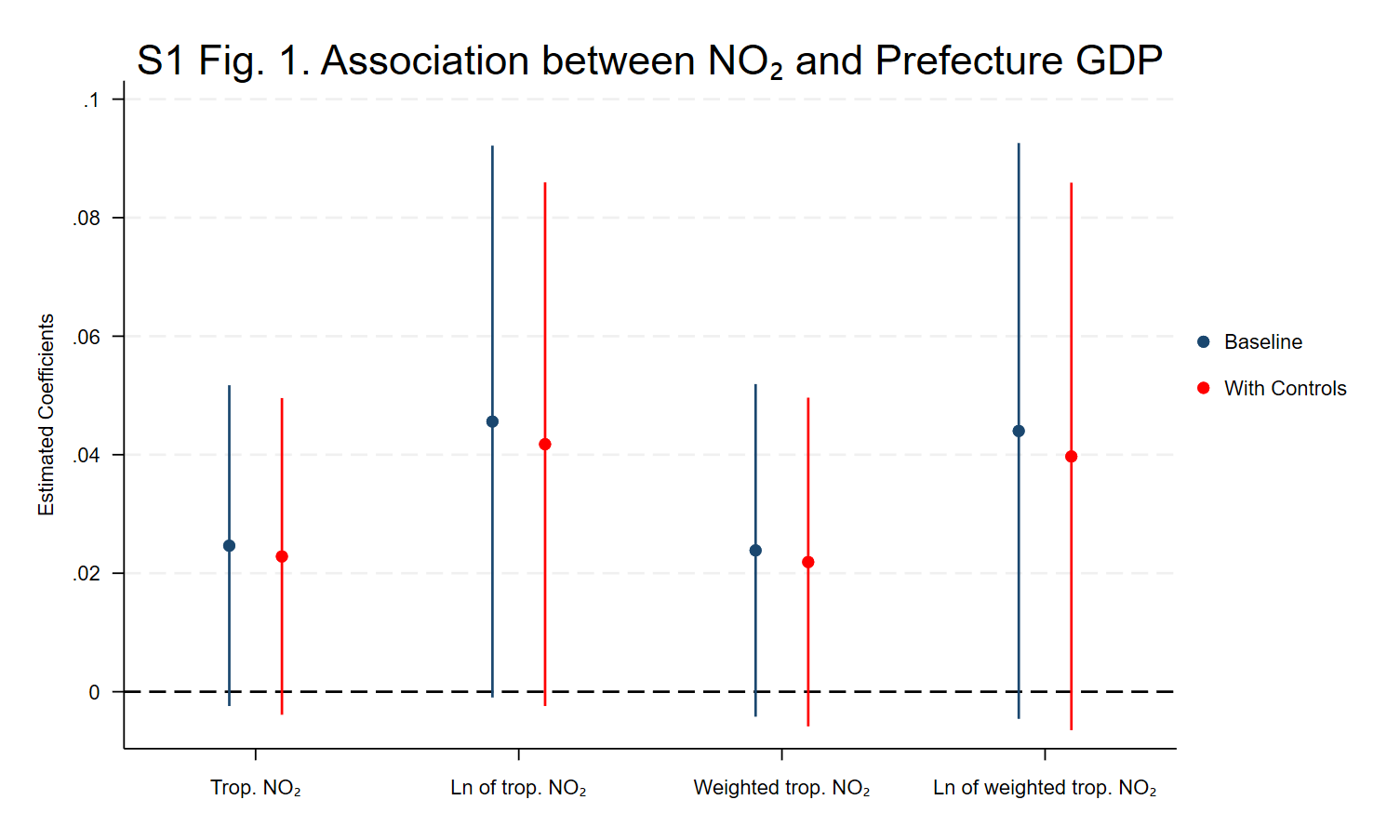
**

*Notes: This figure shows estimated coefficients from fixed effects regressions of log prefecture-level GDP on various measures of tropospheric NO₂ concentration measured at 0.25-degree spatial resolution. Dots represent point estimates and vertical lines show 90% confidence intervals. Navy dots and lines indicate baseline specifications without precipitation and temperature controls; red dots and lines indicate specifications controlling for average precipitation and average temperature. All specifications include prefecture and year fixed effects. Standard errors are clustered at the prefecture level.*

**S1 Fig. B. Baseline and Precipitation- and Temperature-Controlled Estimates: Association between NO₂ and Mining Sector GDP.**

**
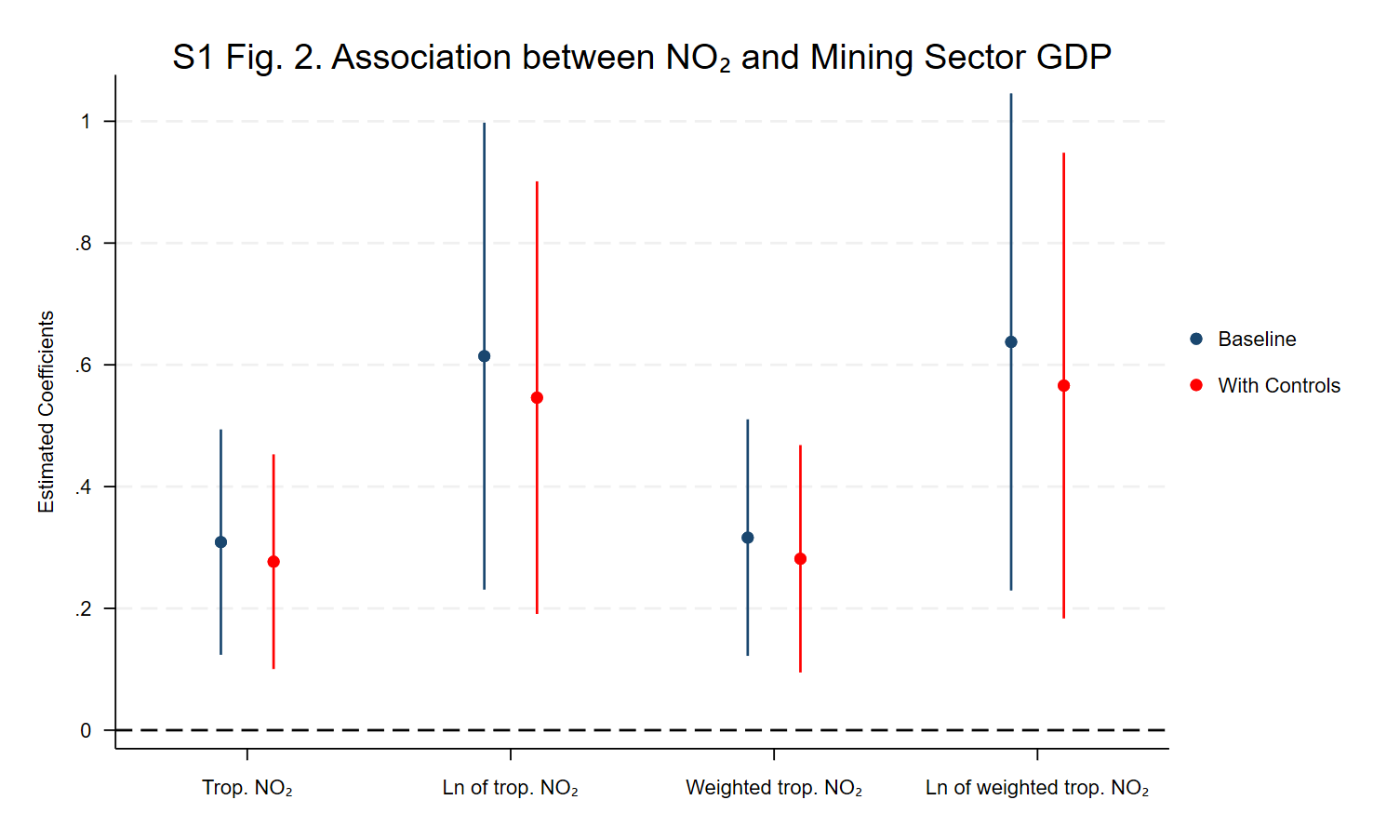
**

*Notes: This figure shows estimated coefficients from fixed effects regressions of natural logarithm mining sector GDP on various measures of tropospheric NO₂ concentration measured at 0.25-degree spatial resolution. Dots represent point estimates and vertical lines show 90% confidence intervals. Navy dots and lines indicate baseline specifications without precipitation and temperature controls; red dots and lines indicate specifications controlling for average precipitation and average temperature. All specifications include prefecture and year fixed effects. Standard errors are clustered at the prefecture level.*

**S1 Fig. C. Baseline and Precipitation- and Temperature-Controlled Estimates: Association between NO₂ and Electricity, Gas and Water Sector GDP.**

**
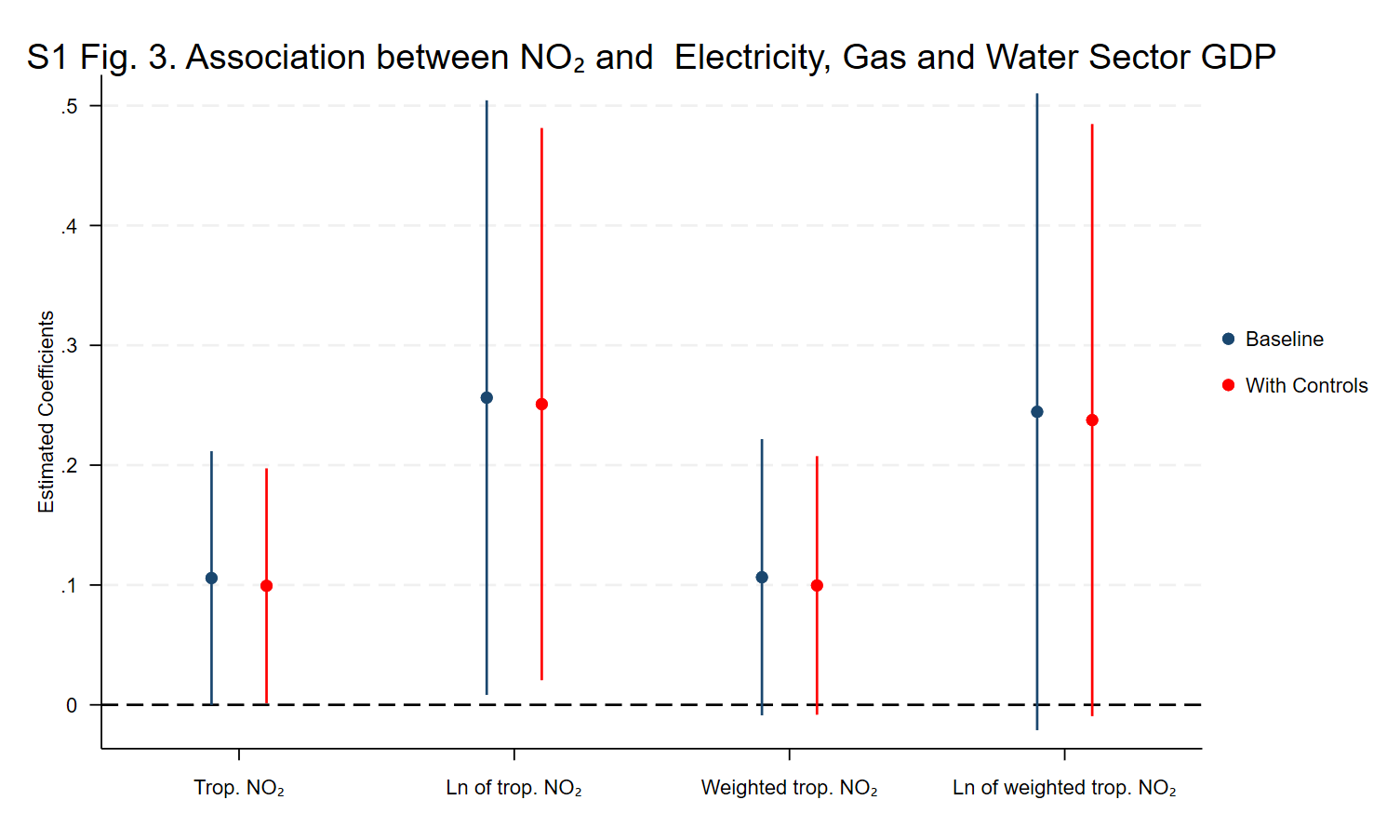
**

*Notes: This figure shows estimated coefficients from fixed effects regressions of natural logarithm electricity, gas and water sector GDP on various measures of tropospheric NO₂ concentration measured at 0.25-degree spatial resolution. Dots represent point estimates and vertical lines show 90% confidence intervals. Navy dots and lines indicate baseline specifications without precipitation and temperature controls; red dots and lines indicate specifications controlling for average precipitation and average temperature. All specifications include prefecture and year fixed effects. Standard errors are clustered at the prefecture level.*

**S1 Fig. D. Baseline and Precipitation- and Temperature-Controlled Estimates: Association between NO₂ and Agriculture, Foresty and Fisheries Sector GDP.**

**
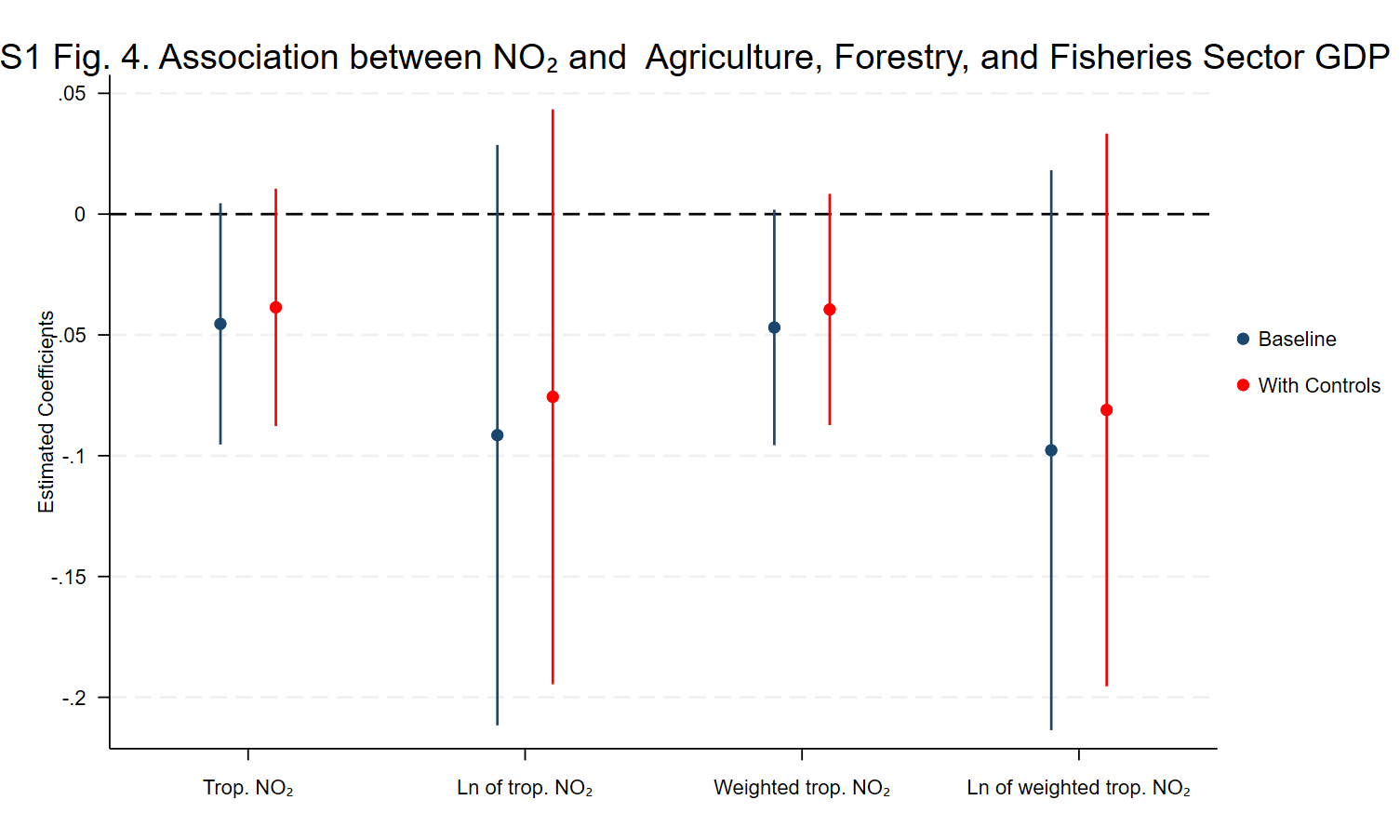
**

*Notes: This figure shows estimated coefficients from fixed effects regressions of natural logarithm agriculture, forestry, and fisheries sector GDP on various measures of tropospheric NO₂ concentration measured at 0.25-degree spatial resolution. Dots represent point estimates and vertical lines show 90% confidence intervals. Navy dots and lines indicate baseline specifications without precipitation and temperature controls; red dots and lines indicate specifications controlling for average precipitation and average temperature. All specifications include prefecture and year fixed effects. Standard errors are clustered at the prefecture level.*

**S1 Fig. E. Baseline and Precipitation- and Temperature-Controlled Estimates: Association between NO₂ and Construction Sector GDP.**

**
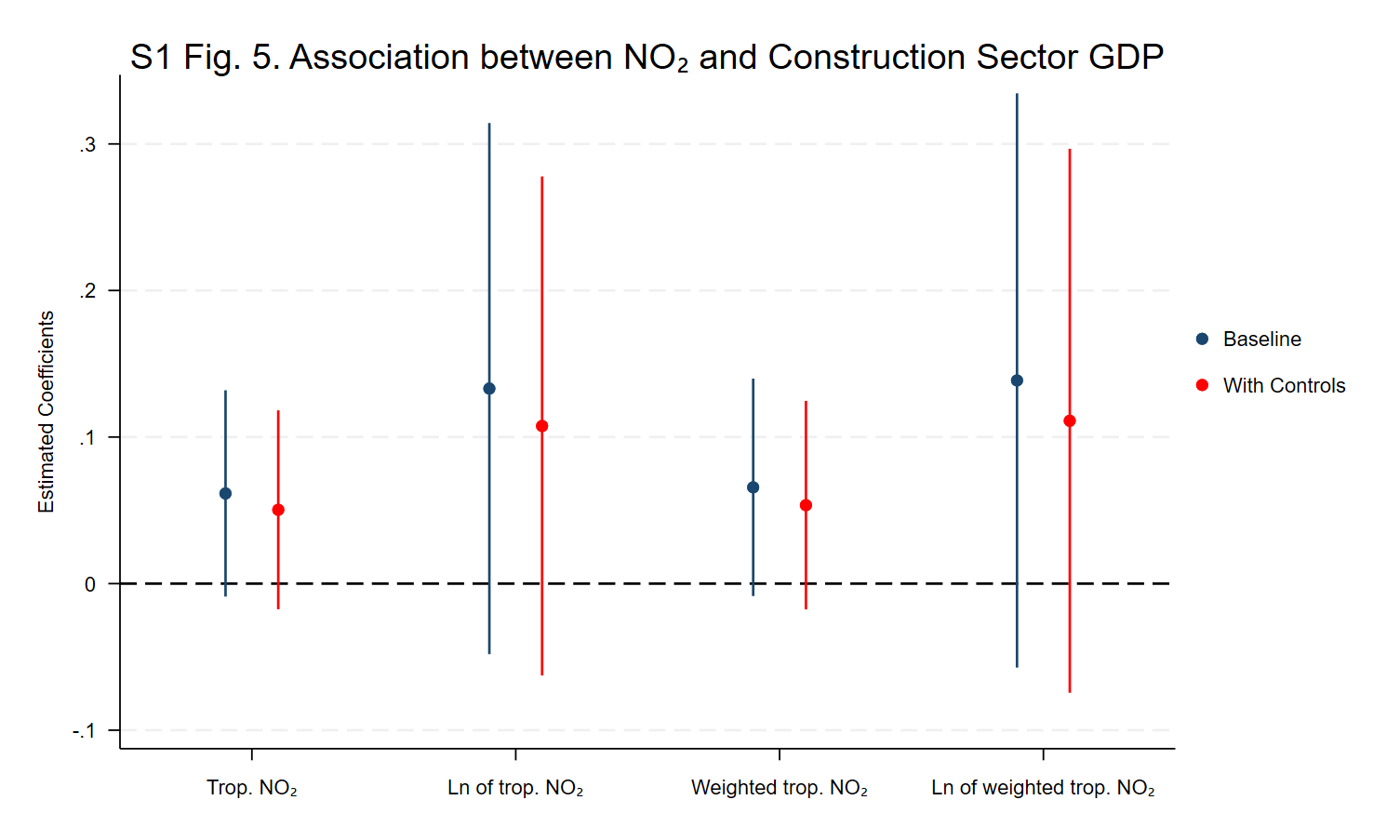
**

*Notes: This figure shows estimated coefficients from fixed effects regressions of natural logarithm construction sector GDP on various measures of tropospheric NO₂ concentration measured at 0.25-degree spatial resolution. Dots represent point estimates and vertical lines show 90% confidence intervals. Navy dots and lines indicate baseline specifications without precipitation and temperature controls; red dots and lines indicate specifications controlling for average precipitation and average temperature. All specifications include prefecture and year fixed effects. Standard errors are clustered at the prefecture level.*

**S1 Fig. F. Baseline and Precipitation- and Temperature-Controlled Estimates: Association between NO₂ and Manufacturing Sector GDP.**

**
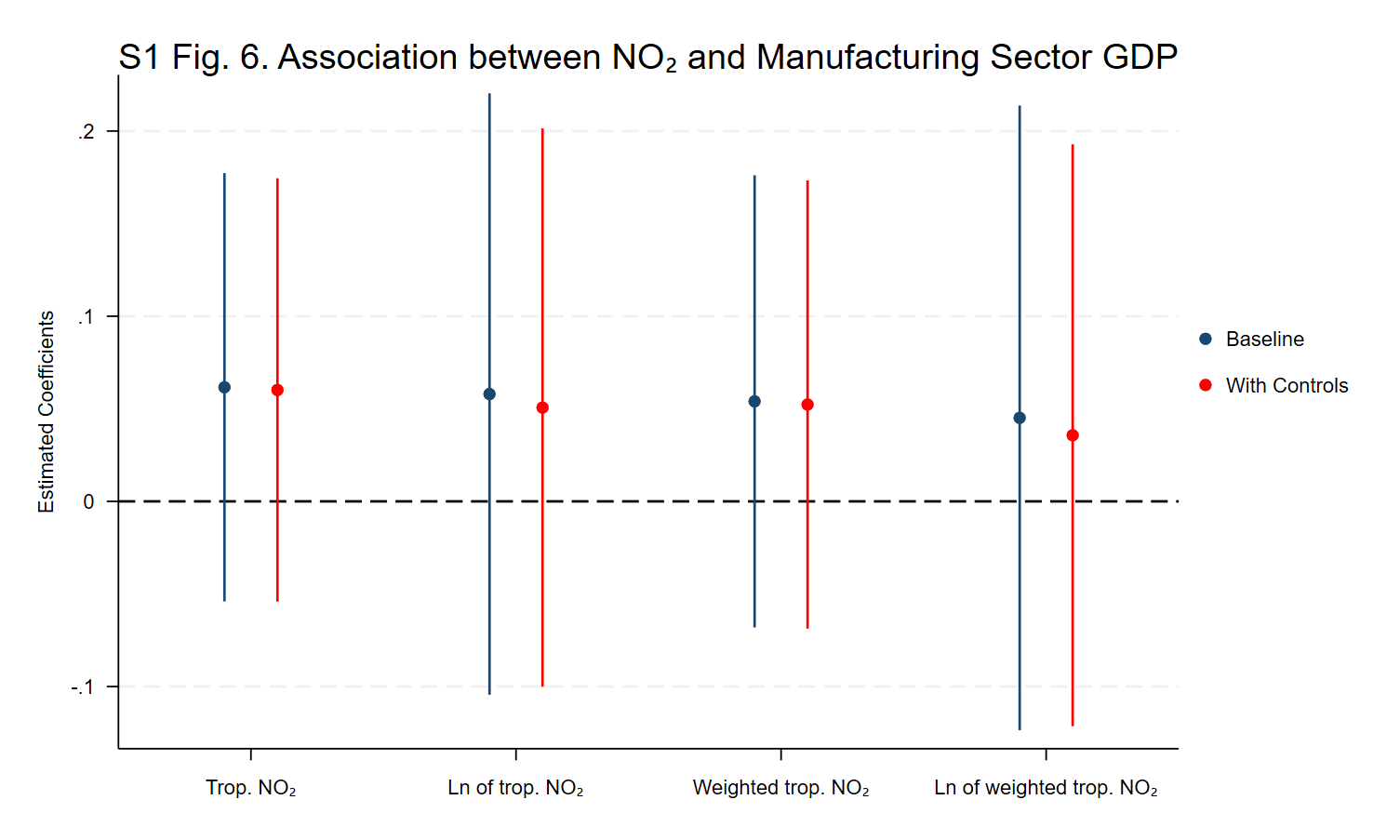
**

*Notes: This figure shows estimated coefficients from fixed effects regressions of natural logarithm manufacturing sector GDP on various measures of tropospheric NO₂ concentration measured at 0.25-degree spatial resolution. Dots represent point estimates and vertical lines show 90% confidence intervals. Navy dots and lines indicate baseline specifications without precipitation and temperature controls; red dots and lines indicate specifications controlling for average precipitation and average temperature. All specifications include prefecture and year fixed effects. Standard errors are clustered at the prefecture level.*
